# Adherence, exposure and patients’ experiences with the use of erlotinib in non-small cell lung cancer

**DOI:** 10.1007/s00432-015-1935-0

**Published:** 2015-03-06

**Authors:** Lonneke Timmers, Christel C. L. M. Boons, J. Moes-ten Hove, Egbert F. Smit, Peter M. van de Ven, Joachim G. Aerts, Eleonora L. Swart, Epie Boven, Jacqueline G. Hugtenburg

**Affiliations:** 1 Department of Clinical Pharmacology and Pharmacy, VU University Medical Center, De Boelelaan 1117, 1081 HV Amsterdam, The Netherlands; 2 Department of Pulmonology, VU University Medical Center, Amsterdam, The Netherlands; 3 Department of Epidemiology and Biostatistics, VU University Medical Center, Amsterdam, The Netherlands; 4 Department of Pulmonology, Erasmus University Medical Center, Rotterdam, The Netherlands; 5 Department of Pulmonology, Amphia Hospital Breda, Breda, The Netherlands; 6 Department of Medical Oncology, VU University Medical Center, Amsterdam, The Netherlands; 7 The EMGO Institute for Health and Care Research, VU University Medical Center, Amsterdam, The Netherlands

**Keywords:** Erlotinib, Non-small cell lung cancer, Medication adherence, Symptoms, Patient-reported, Tyrosine kinase inhibitor

## Abstract

**Purpose:**

Erlotinib is an orally administered tyrosine kinase inhibitor used for treatment of non-small cell lung cancer. Understanding actual use of medication is essential for optimizing treatment conditions.

**Methods:**

In this multicentre prospective observational study, patients starting erlotinib treatment were followed for 4 months. Adherence was assessed using a medication event monitoring system (MEMS). Area under the curve (AUC) was determined after 1, 2 and 4 months. Before start and at monthly intervals, patients filled out questionnaires about attitude towards medication and disease, quality of life, symptoms and use in daily practice.

**Results:**

Sixty-two patients (median age 63.5 years, 53 % male) were included of whom 15 were still on treatment after 4 months. MEMS data of 55 patients revealed a mean adherence of 96.8 ± 4.0 %. Over one-third of patients had an adherence rate <95 %. At 1 month, 21 % of patients did not always correctly take erlotinib without food. Associated risk factors were older age, suboptimal adherence, ocular symptoms and stomatitis (all *p* < 0.05). After 1 month of treatment, fatigue (91 %) and rash (86 %) were the most common symptoms reported. AUC_ss_ of erlotinib was higher in patients with rash and patients with moderate–severe anorexia (both *p* < 0.05).

**Conclusion:**

Though adherence to erlotinib treatment is generally high, non-adherence might be an issue in a considerable number of patients. To support optimal erlotinib intake, clinicians need to take adequate measures to ameliorate symptoms and to address adherence and correct intake without food. Especially older patients and those who experience stomatitis may need extra attention.

## Introduction

Erlotinib is an orally administered tyrosine kinase inhibitor (TKI) of the epidermal growth factor receptor (EGFR) indicated as first-line treatment for advanced non-small cell lung cancer (NSCLC) in patients with activating EGFR mutations and as second- and third-line treatment in patients with EGFR wild-type NSCLC. In the pivotal study, erlotinib prolonged survival, reduced tumour-related adverse events and improved quality of life (Shepherd et al. 2005; Bezjak et al. 2006). Response to erlotinib is highly dependent on the mutation status of EGFR in tumour tissue (Cadranel et al. 2012; Petrelli et al. 2012a). The prognosis is particularly poor in patients with EGFR wild-type, while substantially better in patients with EGFR mutations treated first line with erlotinib (Petrelli et al. 2012a). The most common adverse events of erlotinib occurring in >10 % of patients are skin rash, anorexia, diarrhoea, (kerato) conjunctivitis and fatigue (EMA 2014; Shepherd et al. 2005). In another meta-analysis of 24 trials in which the EGFR mutation status was not taken into account, skin rash was found to be an independent predictive factor for survival and progression (Petrelli et al. 2012b). In addition, patients who developed grade 2–4 rash had a higher chance to respond to treatment as compared to patients without rash (Petrelli et al. 2012b). Dose increase to achieve rash in individual patients, however, has resulted in controversial findings (Mita et al. 2011; Brahmer et al. 2014). From a patient’s view, drug-induced symptoms may induce considerable physical and psycho-social discomfort, which in daily practice are known to affect medication adherence (Verbrugghe et al. 2013; Sabaté 2003). Non-adherence may lead to suboptimal clinical outcomes and increased healthcare costs (Sabaté 2003).

Adherence rates of patients using oral anti-cancer agents have been shown to range from less than 20 to 100 % (Partridge et al. 2002; Ruddy et al. 2009). Understanding of patients’ actual use of medication is essential for creating treatment conditions that will provide optimal clinical benefit. Patient-reported outcomes therefore should be taken into consideration in the treatment decision-making process as they better reflect daily health status than adverse event assessments by clinicians (Basch et al. 2009; Basch 2010). At present, little is known about the use of erlotinib in daily practice from the patients’ perspective. This study in patients with advanced or metastatic NSCLC was designed to assess adherence to erlotinib treatment and evaluate experiences of patients as well as the relationship between medication adherence, erlotinib exposure and symptoms (Timmers et al. 2011).

## Patients and methods

### Study design

This prospective observational cohort study (Timmers et al. 2011) was conducted between October 2009 and July 2011 in 12 Dutch hospitals. Patients with advanced NSCLC scheduled for treatment with erlotinib regardless their EGFR mutation status were eligible for participation. Exclusion criteria were: age younger than 18 years or inability to fill out a Dutch questionnaire. Patients starting erlotinib were followed for a maximum of 4 months. Adherence to erlotinib was measured with a medication event monitoring system (MEMS). Blood samples were collected after 1, 2 and 4 months. Patients filled out questionnaires before the start of erlotinib and at monthly intervals on treatment. The study was approved by the Medical Ethics review board of VU University Medical Center (VUMC, Amsterdam, The Netherlands), as well as the Medical Ethics review board of each participating centre. Written informed consent was obtained from all patients.

### Data collection

#### Medication adherence

Adherence was measured using MEMS (SIMpill^®^, Evalan, Amsterdam, The Netherlands). This pill box contains an electronic processor that records all time-points the box is opened. MEMS data were used for observational purposes only. Only MEMS data from patients who used erlotinib >7 days were included for this analysis. Adherence was expressed as the proportion of days covered (PDC) and was calculated by the number of days on which the box was opened divided by the total number of days of treatment (for a maximum of 4 months) × 100 %. To calculate the proportion of days with multiple openings (PDMO), the number of days on which the box was opened at least twice was divided by the total number of days of treatment (for a maximum of 4 months) × 100 %. Multiple openings on the first and last day of use were not considered for calculation of PDMO.

#### Erlotinib AUC

A steady-state blood sample was collected at regular visits after 1, 2 and 4 months of treatment in those patients taking erlotinib 150 mg/day. The time of blood withdrawal and the time of the last ingestion of erlotinib before blood sampling were registered. Samples were centrifuged, and the plasma was stored at −20 °C until analysis.

Plasma concentrations of erlotinib were analysed by a validated liquid chromatography MS/MS method. The lower limit of quantification (LLOQ) was set at 7 ng/ml. For post hoc estimation of the area under the curve (0–24 h) at steady state (AUC_ss_), the data were analysed with the NONMEM program (version VII, level 2 ICON), using a one-compartment first-order absorption and elimination model adapted from the model described by Lu et al. (2006). The bioavailability F was set at 1. The typical population values for the parameters: absorption constant, Ka (0.89 h^−1^), clearance (4.29 L/h) and volume of distribution V (210 L) were set according to the publication of Lu et al. Covariates (total bilirubin, α1-acid glycoprotein, smoking) in the model were tested with backward elimination for our population. Selection of the covariates of the model was based on the likelihood ratio test. The objective function value (OFV) is proportional to −2 times the log likelihood of the data, given the model; therefore, the difference between two hierarchic models in OFV (ΔOFV) is approximately *χ* ^2^-distributed. A ΔOFV between the competing models of 3.84 (d*f* = 1), corresponding to a nominal *p* value of <0.05, was required for the more complex model to be regarded as significantly better than the less complex one. After the model was selected, systemic exposure to erlotinib as AUC_ss_ was calculated.

#### Questionnaires

At baseline, patients were asked to fill out questionnaires including demographic characteristics, smoking, co-medication (including acid-inhibiting medication), quality of life [Short Form-12 Health Survey (SF-12)] (Aaronson et al. 1998; Gandek et al. 1998), attitude towards medication in general and specifically towards erlotinib (Beliefs about Medicines Questionnaire [BMQ]) (Horne et al. 1999), illness perception (Brief Illness Perception Questionnaire [Brief IPQ]) (Broadbent et al. 2006) and symptoms. After each month of treatment, patients filled out questionnaires about adherence behaviour (Medication Adherence Report Scale [MARS]) (Butler et al. 2004; Horne et al. 2001), overuse, erlotinib intake in relation to food intake, quality of life, illness perception, patients’ attitude towards medication in general and specifically towards erlotinib and symptoms. Intake of erlotinib in relation to food intake was scored as incorrect when patients reported taking erlotinib <60 min before or <120 min after food intake. The questions on symptoms were distracted from the literature on erlotinib toxicities occurring in >10 % of patients, and answers were scored on a 5 point Likert scale (not at all, a little bit, rather, a lot, very much). The scores at baseline and the scores during use were considered as, respectively, baseline health problems and patient-reported symptoms. Patient-reported symptoms scored as ‘a lot’ or ‘very much’ were considered as ‘severe’. Patients taken off treatment before the end of the four-month follow-up period were asked to fill out questions about reasons for discontinuation.

#### Patients’ medical file

Information on disease characteristics and dose adjustment or interruption by the physician was derived from the patients’ medical file.

### Statistics

Baseline descriptive data were analysed as frequencies (percentages) for categorical variables and as the mean (±standard deviation) for continuous data. Adherence over time was summarized by mean PDC and mean PDMO per time-period. Associations between baseline characteristics and incorrect use of erlotinib separately from food were assessed in univariate logistic regression analyses in which incorrect use was taken as the dependent variable. Associations between repeatedly measured variables and incorrect intake of erlotinib over time were tested using generalized estimating equation analyses (GEE). The nonparametric Mann–Whitney test was used to compare the AUC of patients with and without symptoms and patients using and not using acid-inhibiting medication (i.e. proton-pump inhibitors or histamine H2-receptor antagonists) at one month. For the comparison between patients with or without symptoms, two separate analyses were performed using different cut-off points: ‘not at all’ versus ‘any symptom’, and ‘not at all’ or ‘a little bit’ (no and mild) versus ‘rather’, ‘a lot’, or ‘very much’ (moderate and severe). The Fisher exact test was used to compare patient-reported symptoms from the present study with adverse events as reported in the BR.21 clinical trial using the Common Terminology Criteria for Adverse Events (CTCAE) system (vs. 2) (Shepherd et al. 2005). For all analyses, a two-tailed significance level of 0.05 was used. *p* values below this level were considered statistically significant. The statistical analyses were performed with SPSS 20.0 for Windows (IBM Corp, Armonk, NY, USA).

## Results

### Baseline characteristics

A total of 62 patients (median age 63.5 years; 53 % male) were included, of whom 15 were still on treatment after 4 months. With the exception of 2 patients, all patients started with a once daily dose of 150 mg. Thirteen (22 %) dropped out before the measurement at 1 month and 33 (53 %) before 2 months. Table 1 provides information on the number of patients for which data are available at specific time-points. The median duration of treatment of the 62 patients was 51.5 days, with a range from 2 days to the maximum of the observation period of 120 days. Baseline characteristics of the patients are shown in Table 2.

**Table 1:** Number of patients for which data are available at time-point

	*T*0	*T*1	*T*2	*T*3	*T*4
Patients on treatment	62	49	29	17	15
Adherence MEMS	55	45	27	16	14
Blood sample	x	42	28	x	13
Questionnaire	62	47	27	17	14
MEMS + blood sample	x	39	26	x	12
MEMS + questionnaire	55	44	25	16	13
Blood sample + questionnaire	x	41	26	x	12
MEMS + blood sample + questionnaire	x	39	24	x	11

MEMS, medication event monitoring system; T0, baseline; T1, T2, T3 and T4 represent time-points 1, 2, 3 and 4 months

**Table 2:** Baseline characteristics

	*n* ^a^	%
Age median (years)	62	63.5
Range	46–80	
Male	62	53.2
Low education	60	40.0
Living alone	62	14.5
Paid work	58	17.2
Smoking	62	19.4
Co-morbidity	61	65.6
Co-medication	62	82.3
Number of drugs	51	
Mean ± SD	5.0 ± 2.8	
Range	1–13	
Initial erlotinib dose 150 mg	60	98.3
Duration of use (days)	62	
Mean ± SD	58.3 ± 39.4	
Range	2–120^b^	

*SD* standard deviation

^a^Number of patients with data available

^b^Maximum observation period was 120 days

### Medication adherence

Most patients (55/62, 89 %) used MEMS during the observation period. Two patients refused to use MEMS, three patients used erlotinib <7 days and the MEMS data of another 2 patients were not evaluable due to technical problems. The mean PDC during the observation period was 96.8 ± 4.0 %, with a range from 85 to 100 % (Table 3). Over one-third of patients (19/55) covered <95 % of days. Thirty-four patients (62 %) had at least one day with multiple openings. The mean PDMO of these patients during the studied period was 5.6 ± 4.8 %, with a range from 1 to 21 %. The mean adherence measured with MARS (scale 5–25) was 24.9 ± 0.4 after 1 month and remained above 24 on the other time-points. The MARS statements showed 3 patients (5 %) rarely forgetting to take their erlotinib, 1 patient (2 %) rarely adjusting the dose and 1 patient (2 %) temporarily stopping. 1 patient (2 %) reported to rarely use more than prescribed.

**Table 3:** Adherence to erlotinib as measured with MEMS

	*n*	Mean ± SD	Median	Range
Duration of use (days)	55	60.2 ± 38.8	52.0	6.0–120.0
PDC	55	96.8 ± 4.0	98.0	85.0–100.0
PDC (period 0–28 days)	55	97.5 ± 5.0	100.0	71.4–100.0
PDC (period 29–56 days)	39	96.5 ± 6.4	100.0	71.4–100.0
PDC (period 57–max 120 days)	25	95.2 ± 6.7	100.0	75.0–100.0
PDMO	34	5.6 ± 4.8	4.1	1.0–21.2

		%
100 % PDC	55	43.6
≥95 % PDC	55	65.5
≥90 % PDC	55	92.7

*PDC* proportion of days covered, *PDMO* proportion of days with multiple openings, *SD* standard deviation, *MEMS* medication event monitoring system

### Quality of life and patients’ beliefs and attitude

The mean score at baseline of the SF-12 physical component was 32.5 ± 9.3, and the SF-12 mental component was 47.7 ± 8.2. The BMQ subscales were 10.8 ± 2.6 (overuse), 10.3 ± 2.3 (harm), 19.1 ± 3.2 (necessity), 15.3 ± 4.0 (concerns) and 4.0 ± 4.5 (differential). The classification into 4 attitudinal groups based on their responses to the BMQ at baseline was: 40.0 % accepting (high necessity, low concerns), 55.0 % ambivalent (high necessity, high concerns), 3.3 % indifferent (low necessity, low concerns) and 1.7 % sceptical (low necessity, high concerns). The mean scores of Brief IPQ at baseline were 8.0 ± 1.9 (consequences), 8.5 ± 2.5 (time line), 4.0 ± 2.8 (personal control), 6.4 ± 2.3 (treatment control), 7.9 ± 2.3 (identity), 6.0 ± 2.8 (concern), 5.7 ± 3.3 (understanding) and 5.4 ± 2.9 (emotional response).

### Symptoms

Patient-reported symptoms are listed in Table 4. The most reported symptoms at baseline were fatigue (89 %), emotional symptoms (anger, fear, depression and/or mood swings) (74 %), dyspnoea (71 %) and cough (70 %). After 1 month of treatment, fatigue (91 %), rash (86 %), cough (77 %), anorexia (74 %), emotional symptoms (67 %), dyspnoea (66 %) and diarrhoea (66 %) were the symptoms most commonly reported. Fatigue was the symptom most commonly occurring at baseline (89 %) and during treatment (91, 83, 88 and 85 % at, respectively, 1, 2, 3 and 4 months). Severe symptoms most commonly reported after 1 month of treatment were rash (39 %), fatigue (32 %), sleeping problems (29 %) and anorexia (21 %).

**Table 4:** Patient-reported symptoms

*n*	% any					% severe^a^					% increase^b^			
	*T*0	*T*1	*T*2	*T*3	*T*4	*T*0	*T*1	*T*2	*T*3	*T*4	*T*1	*T*2	*T*3	*T*4
	62	49	29	17	15	62	49	29	17	15	49	29	17	15
Fatigue	88.5	90.9	82.6	88.2	84.6	26.2	31.8	17.4	17.6	23.1	21.7	52.2	58.8	53.8
Rash	14.8	81.8	87.0	82.4	84.6	3.3	38.6	30.4	29.4	38.5	81.8	81.8	81.3	91.7
Anorexia	54.8	70.5	60.9	52.9	61.5	8.1	20.5	21.7	23.5	15.4	39.1	39.1	35.3	23.1
Diarrhoea	19.4	65.9	65.2	52.9	76.9	1.6	11.4	13.0	17.6	15.4	56.5	52.2	52.9	69.2
Ocular symptoms	26.2	59.1	39.1	35.3	69.2	0	4.5	4.3	0	0	36.4	22.7	18.8	41.7
Nausea/vomiting	23.0	51.2	30.4	23.5	23.1	6.6	14.6	4.3	5.9	0	33.3	28.6	12.5	8.3
Stomatitis	3.3	22.0	14.3	29.4	38.5	0	7.3	4.8	5.9	7.7	16.7	11.1	31.3	41.7
Infection	1.7	11.6	8.7	17.6	41.7	0	2.3	0	5.9	8.3	0.0	4.5	18.8	36.4
Cough	70.0	76.7	77.3	64.7	53.8	5.0	9.3	9.1	0	23.1	20.0	35.0	12.5	33.3
Dyspnoea	71.0	65.9	60.9	64.7	66.7	9.7	18.2	8.7	17.6	25.0	26.1	39.1	41.2	41.7
Sleep problems	47.5	45.5	39.1	53.3	46.2	13.1	28.6	4.3	13.3	15.4	13.6	13.6	26.7	23.1
Emotional symptoms	74.1	67.4	56.5	70.6	69.2	13.6	13.6	4.3	5.9	15.4	33.3	28.6	40.0	36.4
Stomach ache	23.0	37.2	43.5	47.1	30.8	4.9	2.3	0	0	0	19.0	38.1	37.5	25.0
Headache	31.3	20.5	21.7	11.8	30.8	3.3	0	0	0	0	4.5	9.1	6.3	16.7
Pain when breathing	13.1	14.0	17.4	25.0	15.4	0	0	0	0	7.7	0.0	13.6	13.3	8.3

Missing data excluded from frequency analyses

T0, at baseline; T1, at 1 month; T2, at 2 months; T3, at 3 months; T4, at 4 months

^a^% of patients with symptoms reported as ‘a lot’ or ‘very much’

^b^% of patients with a (any grade) symptom score higher than the score at baseline

Table 5 shows the change in patient-reported symptoms over time for patients with at least 2 months of follow-up.

**Table 5:** Longitudinal description of patient-reported symptoms

(*n* = 29)	*T*0		*T*1		*T*2	
	% any	% severe	% any	% severe	% any	% severe
Fatigue	82.6	25.0	87.0	20.7	82.6	17.4
Rash	9.1	0	86.4	24.7	86.4	30.4
Anorexia	56.5	3.4	73.9	13.8	60.9	21.7
Diarrhoea	17.4	0	69.6	10.3	65.2	13.0
Ocular symptoms	22.7	0	54.5	3.4	36.4	4.3
Nausea/vomiting	19.0	7.1	38.1	10.7	28.6	4.3
Stomatitis	0.0	0	16.7	3.7	11.1	4.8
Infection	0.0	0	0.0	0	4.5	0
Cough	70.0	7.4	75.0	7.1	75.0	9.1
Dyspnoea	65.2	6.9	52.2	10.3	60.9	8.7
Sleep problems	50.0	10.7	40.9	10.3	40.9	4.3
Emotional symptoms	76.2	7.7	57.1	10.3	57.1	4.3
Stomach ache	4.8	0	23.8	3.6	42.9	0
Headache	31.8	0	22.7	3.4	18.2	0
Pain when breathing	18.2	0	4.5	0	13.6	0

T0, at baseline; T1, at 1 month; T2, at 2 months

Patient-reported symptoms mentioned after 1 month (*n* = 49) were compared with adverse events observed in the pivotal phase III BR.21 study (Shepherd et al. 2005) (Table 6). Though not significantly different from the toxicities reported in BR.21 (*p* > 0.05), the occurrence of most patient-reported symptoms (fatigue, rash, anorexia, diarrhoea, nausea/vomiting and stomatitis) was higher than the adverse events scored by clinicians in the BR.21 study. Ocular symptoms were reported more frequently by patients in the present study and infection less often as compared to BR.21 (*p* < 0.05).

**Table 6:** Patient-reported symptoms versus clinical trial adverse events**

*T*1	PRO		Clinical trial^a^		*p* value
	*n*	%	*n*	%	
Fatigue	44	90.9	485	79	0.075
Rash	44	81.8	485	76	0.461
Anorexia	44	70.5	485	69	0.057
Diarrhoea	44	65.9	485	55	0.205
Ocular symptoms	44	59.1	485	28	<0.001*
Nausea/vomiting	41	51.2	485	40	0.186
Stomatitis	41	22.0	485	19	0.679
Infection	43	11.6	485	34	<0.001*

*PRO* patient-reported outcomes

* Significant; T1, at 1 month; ** symptoms scored by patients in this study and adverse events scored by clinicians in the BR.21 trial (CTCAE vs. 2) independent of severity

^a^BR.21 trial (Shepherd et al. 2005)

### Adherence and use in daily practice

Most patients (>70 %) reported to use any reminder method to support the erlotinib intake. None of the patients reported the intake of grapefruit or grapefruit juice during treatment with erlotinib. No relationship was found between adherence (assessed with MEMS) and the use of a reminder method. After 1 month, 21 % of patients did not always correctly take erlotinib without food. Tables 7 and 8 show the risk factors of not always taking erlotinib under fasting conditions. Significant relationships with incorrect intake were: older age (OR 1.10, 95 % CI 1.00–1.21), MARS < 25 (OR 4.83, 95 % CI 1.06–21.99), oculair symptoms (OR 3.13, 95 % CI 1.11–8.82) and stomatitis (OR 6.59, 95 % CI 1.77–24.60).

**Table 7:** Relations with incorrect intake of erlotinib under fasting conditions^a^

*N* = 41	OR	95 % CI	*p* value
Male	1.20	0.30–4.80	0.797
Age	1.10	1.00–1.21	0.041*
Low education	3.00	0.69–13.12	0.144
Living alone	0.50	0.05–4.83	0.549
No paid work	2.86	0.31–26.74	0.356
No smoking	1.54	0.15–15.49	0.715
No Co-morbidity	2.80	0.68–11.59	0.155
Number of co-medication	0.96	0.75–1.22	0.717

Missing data excluded from analyses

*OR* odds ratio, *CI* confidence interval

* Significant

^a^Analyses of baseline characteristics using logistic regression

**Table 8:** Relations with incorrect intake of erlotinib under fasting conditions^a^

*N* = 40	OR	95 % CI	*p* value
SF-12			
Physical component	1.00	0.99–1.01	0.959
Mental component	1.02	1.00–1.04	0.079
BMQ			
General overuse	0.97	0.92–1.02	0.279
General harm	0.97	0.92–1.03	0.306
Specific necessity	1.01	0.97–1.04	0.774
Specific concerns	0.99	0.97–1.02	0.593
Necessity-concerns differential	1.00	0.99–1.02	0.564
Brief IPQ			
Consequences	1.01	0.97–1.04	0.691
Time line	1.01	0.96–1.07	0.588
Personal control	0.97	0.94–1.00	0.085
Treatment control	1.03	0.96–1.11	0.374
Identity	1.02	0.97–1.06	0.417
Concern	0.97	0.94–1.01	0.124
Understanding	0.98	0.95–1.02	0.313
Emotional response	0.99	0.96–1.02	0.449
MARS < 25	4.83	1.06–21.99	0.042*
No reminder method	2.79	0.52–14.82	0.229
Patient-reported symptom (any severity)			
Rash	2.52	0.44–14.21	0.295
Anorexia	0.36	0.11–1.20	0.097
Diarrhoea	8.16	0.43–154.09	0.161
Ocular symptoms	3.13	1.11–8.82	0.031*
Nausea/vomiting	0.62	0.13–3.02	0.554
Stomatitis	6.59	1.77–24.60	0.005*
Infection	1.18	0.12–12.02	0.891
Cough	0.32	0.08–1.25	0.102
Dyspnoea	5.69	0.83–38.87	0.076
Sleep problems	0.59	0.15–2.32	0.454
Stomach ache	1.69	0.44–6.50	0.445
Headache	0.95	0.18–4.99	0.956
Pain when breathing	0.49	0.08–3.04	0.443

No OR could be estimated for fatigue and emotional symptoms. Missing data excluded from analyses

*OR* odds ratio, *CI* confidence interval, *SF-12* short form-12 health survey, *BMQ* beliefs about medicines questionnaire, *Brief IPQ* illness perception questionnaire, *MARS* medication adherence report scale

* Significant

^a^Analyses using generalized estimated equations (GEE)

### AUC

The mean AUC_ss_ (μg*h/mL) of erlotinib at 1, 2 and 4 months of treatment was 37.8 ± 15.7 (*n* = 42), 36.8 ± 15.8 (*n* = 28) and 40.5 ± 20.4 (*n* = 13), respectively. The relationship between the AUC and patient-reported symptoms at 1 month is presented in Table 9. The mean AUC of erlotinib was significantly higher in patients who reported rash (any severity) (*p* < 0.05). The comparison of the AUC of erlotinib in patients reporting symptoms at the levels ‘not at all’ or ‘a little bit’ (no and mild) with the AUC in those reporting symptoms at the levels ‘rather’, ‘a lot’, or ‘very much’ (moderate and severe) at 1 month was only significantly different for anorexia. Patients reporting moderate or severe anorexia had a significantly higher AUC than patients reporting no or mild anorexia (44.8 ± 4.7 vs. 33.5 ± 2.7 μg*h/mL, *p* < 0.05). A substantial number of patients (39 %) used an acid-inhibiting drug (a proton-pump inhibitor or a histamine H2-receptor antagonist). The AUC of erlotinib of this patient group did not differ between those who did use and those who did not use acid-inhibiting medication (AUC, respectively, 34.5 ± 13.4 vs. 41.4 ± 19.1 μg*h/mL, *p* = 0.283).

**Table 9:** Relationship between AUC_ss_ and patient-reported symptoms (T1)

(*n* = 41)	No symptom AUC (µg*h/mL)		Symptom AUC (µg*h/mL)		*p* value
Symptom	*n*	Mean ± SD	*n*	Mean ± SD	
Fatigue	4	26.2 ± 3.0	34	39.3 ± 2.8	0.074
Rash	7	27.8 ± 4.7	31	40.2 ± 2.8	0.030*
Anorexia	11	32.4 ± 4.8	27	40.2 ± 3.0	0.082
Diarrhoea	14	35.5 ± 4.4	24	39.4 ± 3.2	0.520
Ocular symptoms	18	39.6 ± 4.6	20	36.4 ± 2.6	0.919
Nausea/vomiting	16	33.6 ± 2.7	19	42.3 ± 4.4	0.286
Stomatitis	28	36.8 ± 2.6	8	43.3 ± 8.4	0.614
Infection	33	38.3 ± 2.9	4	34.8 ± 6.0	0.944
Cough	7	35.2 ± 3.6	30	39.3 ± 3.1	0.747
Dyspnoea	13	32.7 ± 3.4	25	40.7 ± 3.4	0.188
Sleep problems	20	35.6 ± 2.9	18	40.6 ± 4.4	0.534
Emotional symptoms	11	34.3 ± 4.6	26	39.7 ± 3.2	0.256
Stomach ache	23	38.6 ± 3.9	15	36.9 ± 2.9	0.768
Headache	31	37.4 ± 3.0	7	40.3 ± 5.0	0.354
Pain when breathing	31	38.8 ± 3.1	6	36.9 ± 2.5	0.888

Missing data excluded from analyses

AUC_ss_, area under the curve at steady state; T1, at 1 month; SD, standard deviation

* Significant

### Discontinuation

Twenty-six patients filled out the questions on reasons for discontinuation. Thirty-one per cent of them reported side effects as a reason to stop erlotinib treatment, whereas 69 % stopped treatment because of lack of clinical efficacy. Most patients (88 %) discontinued on the initiative of their physician.

## Discussion

NSCLC patients prescribed erlotinib were highly compliant. The mean adherence was 96.8 % ± 4.0.

The high adherence rate might be explained by the life-threatening nature of the disease and the short treatment period. Sub-optimal adherence to oral anticancer medication is more often an issue in long-term treatments, such as adjuvant endocrine treatment prescribed after primary breast cancer or TKI treatment of chronic myeloid leukaemia (Ruddy et al. 2009; Marin et al. 2010; Partridge et al. 2010). The median duration of treatment with erlotinib in the present study was 52 days, mainly driven by lack of clinical efficacy. Treatment duration was grossly comparable with that in unselected patients on erlotinib in the BR.21 trial in whom a progression-free survival was measured of 2.2 months (Shepherd et al. 2005). In our study, erlotinib patients were included without knowledge of the EGFR mutation status in tumour tissue. As erlotinib is now increasingly used in pre-selected patients, the duration of use may increase. This might negatively influence adherence.

In the present study, 93 % of the patients had a PDC of at least 90 %. However, over one-third of all patients used erlotinib in <95 % of the prescribed days. Adherence rates of 80–95 % are generally considered as a minimum to obtain an optimal medication efficacy (Osterberg and Blaschke 2005; Ruddy et al. 2009). Adherence <95 %, measured with the self-report Basel Assessment of Adherence Scale (BAAS), has previously been shown related to poorer rates of response to erlotinib (Gebbia et al. 2013). The considerable number of patients (34.5 %) who had a PDC below 95 % confirms that medication adherence might be a relevant issue for this drug.

A minority of patients (21 %) reported occasionally not to follow the recommendations regarding the intake of erlotinib under fasting conditions. Erlotinib should be swallowed at least 1 h before or 2 h after intake of a meal (EMA 2014). Intake under fasting conditions is necessary because food can substantially increase erlotinib plasma levels (Ling et al. 2008), which may cause undesirable adverse events. The presence of stomatitis was significantly related with sub-optimal intake relative to time-points of a meal (OR 6.59, *p* = 0.005). Stomatitis may influence the eating habits and result in an irregular or complex schedule of separation of medication from food. Sub-optimal adherence (MARS < 25) was also significantly related with sub-optimal intake relative to time-points of a meal (OR 4.83, *p* = 0.042). This can be expected, since incorrect intake relative to time-points of a meal, is an aspect of sub-optimal adherence. Older age (OR 1.10, *p* = 0.041) and ocular symptoms (OR 3.13, *p* = 0.031) might also have contributed to non-adherence. Particularly in case of stomatitis, food intake during the use of erlotinib should be carefully addressed by healthcare professionals, on the one hand, to support patients in correctly taking erlotinib in the absence of food and, on the other hand, to avoid anorexia.

As expected, NSCLC patients experienced a variety of symptoms. At baseline, their physical quality of life score was considerably lower (32.5 ± 9.3) than reported for the general population in the Netherlands (47.9 ± 0.6 for 45- to 64-year-olds; 45.2 ± 10.5 for 65- to 74-year-olds). This also holds for the mean mental score (47.7 ± 8.2 vs. 51.4 ± 9.6 for 45- to 64-year-olds and 52.9 ± 8.6 for 65- to 74-year-olds (Gandek et al. 1998). The distribution of patients across the four BMQ-belief groups in our population shows the majority of patients being ambivalent (high necessity, high concerns). This is different from patients with the diseases asthma, cardiac disease, depression or diabetes, where most patients had an accepting (high necessity, low concerns) attitude towards medication (Tibaldi et al. 2009). The severity of the disease and expected symptoms experienced during treatment might play an important role in the high concerns about the medication.

An important issue to address is the role of patients in the assessment of health problems and symptoms to obtain insight into side effects they experience from drug medication (Basch 2010). Advanced NSCLC patients using erlotinib report many health problems, which may not only be caused by their medication but also result from their disease and its progress. In oncology, the collection of data on side effects is clinician based and highly regulated by the CTCAE system (Trotti et al. 2007). A comparison of patient-reported symptoms which occurred in the present real-world study with adverse event data from the clinical trial on erlotinib in NSCLC patients confirms the usefulness of patient-reported outcomes (Basch et al. 2009, 2012; Basch 2010). Although most symptoms did not significantly differ from those scored by clinicians, especially symptoms in the less specific domain were reported more often by patients. Patients’ experiences should always be taken into account, since one-third of the patients reported symptoms as one of the reasons to discontinue treatment. This finding is in line with the results of a previous study in patients using oral anticancer agents (Timmers et al. 2014). Since 88 % of patients discontinued on the initiative of their physician, symptoms reported by the patient may very well have coincided with lack of benefit from erlotinib.

The mean AUC of erlotinib was higher in patients who reported rash (any severity), *p* < 0.05. The relationship between erlotinib exposure and skin rash has been demonstrated previously (Lu et al. 2006; Rudin et al. 2008; Thomas et al. 2009), though the absence of a link between exposure and rash was found as well (Mita et al. 2011). Skin rash is a sign used in clinical practice as indicative for clinical benefit since patients with a high grade of skin rash may experience a longer progression-free and overall survival (Krawczyk et al. 2013; Perez-Soler et al. 2004; Petrelli et al. 2012b; Wacker et al. 2007), though dosing till rash to optimize efficacy is controversial (Mita et al. 2011; Brahmer et al. 2014).

Nowadays, erlotinib is prescribed preferentially for treatment of selected NSCLC patients. The presence of activating EGFR gene mutations and high EGFR gene copy number are predictors of an improved clinical outcome, while the role of KRAS mutations still needs to be elucidated (Zhu et al. 2008; Cadranel et al. 2012). Currently, it is advised to assess the EGFR status before starting first-line treatment with erlotinib (Brugger et al. 2011; Califano et al. 2012). The use of erlotinib in second-line treatment of EGFR wild-type NSCLC patients is an ongoing debate as erlotinib seems less effective than docetaxel (Garassino et al. 2013). With a better understanding of the presence of genetic mutations as a prerequisite for response, the need to use skin rash as a predictor for clinical outcome has become of less importance.

Apart from rash, the mean AUC of erlotinib was also higher in patients reporting moderate or severe anorexia. For other symptoms, such as mucositis, diarrhoea, ocular symptoms and fatigue, frequently reported with the use of erlotinib (Table 4), there was no apparent relationship with the AUC. The use of acid-inhibiting co-medication (i.e. proton-pump inhibitors and/or histamine H2-receptor antagonists) resulted in a lower mean AUC in the present study, though this effect was not significant. It is recommended not to combine erlotinib with gastric acid-suppressive medication because it can reduce bioavailability (EMA 2014). However, a recently performed retrospective analysis of the BR.21 trial database to evaluate the clinical impact of this interaction showed that acid-inhibiting co-medication had no significant impact on plasma levels or outcome (Hilton et al. 2013).

The present study has some strengths and limitations. It provides a unique and complete survey of the use of erlotinib from the patients’ perspective. To our knowledge, adherence to erlotinib neither has been studied previously, nor have real-life data, erlotinib blood samples, patients’ experiences, data retrieved from the patient’s medical file and pharmacies, and adherence assessed with MEMS has been combined in a single study. Unfortunately, the number of included patients is limited. The study was performed in an unselected NSCLC patient population, resulting in a relatively short observation period.

## Conclusion

Medication adherence to erlotinib is an issue in a considerable number of patients with advanced NSCLC. Patients report to have many health problems at baseline and, as to be expected, new symptoms appear during erlotinib treatment. To support patients’ optimal erlotinib intake, clinicians need to take adequate measures to ameliorate symptoms and to address medication adherence and correct intake without food. Especially older patients and those who experience stomatitis may need extra attention to support a correct erlotinib use in daily practice.
